# Outcome of Loupe-Assisted Sub-inguinal Varicocelectomy in Infertile Men

**DOI:** 10.5812/numonthly.1623

**Published:** 2012-06-20

**Authors:** Selim S. Abdelrahman, Bayoumy I. Eassa

**Affiliations:** 1 Departments of Surgery, Al-Azhar University, Cairo, Egypt; 2 Department of Dermatology, Venereology and Andrology, Al-Azhar University, Cairo, Egypt

**Keywords:** Varicocele, Infertility, Semen

## Abstract

**Background:**

Sub-inguinal varicocelectomy is widely used among surgeons.

**Objectives:**

The aim of this study was to evaluate the outcome of varicocelectomy using a modified microsurgical method, specifically a loupe-assisted method, and its effects on sperm parameters in infertile men.

**Patients and Methods:**

This study was performed in 40 patients who presented with varicocele. All patients had at least a 1-year history of infertility with abnormal semen parameters and varicocele proven by physical examination and confirmed with color Doppler ultrasound. Routine preoperative investigations were performed. Semen analysis and hormonal profiling were also performed and repeated postoperatively for follow-up. Half of the patients (20 patients) were treated by a sub-inguinal approach assisted by loupe magnification (Group A) and the other half was treated by the same approach but without magnification (Group B). To facilitate the procedure, an ×3.0 loupe was used during the spermatic cord dissection at the level of the external inguinal ring. During dissection, the dilated veins, including the vassal veins and external spermatic veins, were ligated and divided.

**Results:**

In total, 40 patients were followed for more than 6 months. The age of the patients varied from 25 to 38 years (mean 32.5). No intra-operative complications occurred in both groups. Regarding post-operative complications, Group A contained only one patient (5%) who developed scrotal hematoma and two (10%) who developed wound infection, whereas in Group B, the complication rate was higher: two patients (10%) developed scrotal hematoma, two patients (10 %) developed wound infection, three patients (15%) developed hydrocele, two patients (10%) developed recurrence, and two patients (10%) developed scrotal edema. Regarding the seminal parameters, much improvement was observed in the sperm count and sperm motility, and a decrease in abnormal forms was observed after surgery with significant differences in Group A. In Group B, similar effects were observed, but without significant differences.

**Conclusions:**

Loupe-assisted sub- inguinal varicocelectomy is a safe, simple, and effective method for the treatment of sub-fertile men, especially in medical facilities without microscopic equipment, and permits significant improvement in sperm parameters.

## 1. Background

Varicocele is characterized by the elongation, dilatation and abnormal kinking of the spermatic veins of the pampiniform plexus of the testis (1). Celsius, the Greek physician, mentioned this disease for the first time as testicular atrophy in the first century AD (2). The incidence of varicocele in the male population is 15–20% (3). The fraction of clinically evidenced varicocele in young adult subjects varies from 9% to 23%, as reported by the most recent studies. Furthermore, varicocele is observed in over 40% of infertile persons (4, 5). Approximately 12–25% of men being examined for infertility have moderate to large varicocele, and approximately 15% have small or sub-clinical varicocele (6, 7).

Various studies have demonstrated inconsistent and contradictory results that have led physicians to dissociate varicocele and male infertility. Male fertility may be preserved with only a single healthy testis, whereas infertility represents bilateral testicular dysfunction, so it is difficult to explain bilateral testicular dysfunction with left sided varicocele (8). There are two primary reasons for the controversy regarding the role of varicocelectomy in the management of infertility. First, there is no well-defined etiologic mechanism of varicocele affecting spermatogenesis. It has been postulated that the harmful effect of varicocele in spermatogenesis caused by an increase in the intrascrotal temperature but the exact pathogenic mechanisms that result from raised temperature have not been clearly defined. The second problem is that no well-designed study has proven the beneficial role of surgery (9, 10).

The role of varicocele in the impairment of testicular function and infertility was investigated in men presenting to infertility clinics and documented by the World Health Organization (WHO). Scrotal pain, testicular atrophy, and infertility without other apparent causes are the common indications for the correction of varicocele, whereas surgery in adolescent varicocele, sub-clinical varicocele and azoospermia remains controversial (11). In varicocele, unrelieved venous stasis interferes with the normal testicular temperature, which is usually maintained at 2–3°C lower than the core body temperature (12). Continuous exposure to high temperature causes sub-fertility by decreasing testicular volume, spermatogenesis, and semen quality as well as increasing the amount of immature sperm in the ejaculate (13).When clinical palpable varicocele coexists with impaired semen quality, surgical repair may potentially restore spermatogenesis and fertility (14-16).

The internal spermatic veins are responsible for the testicular venous drainage, but the failure of varicocele repair may be caused by collateral channels such as external spermatic veins or vassal veins (17, 18) Varicocelectomy requires the meticulous inspection of the spermatic cord and it is of prime importance to identify and avoid injury to the arterial blood supply and lymphatic channels to the testicles (19). Contrary to classical descriptions of testicular arterial anatomy that depict a single testicular artery branching at the level of the scrotum, the surgeon must be well versed with the knowledge of the testicular arterial anatomy during surgery of the spermatic cord and scrotal structures to ensure that testicular function and male fertility potential are preserved (20).

Many investigators using both clinical and histological analyses have documented the presence of multiple arterial branches within the inguinal spermatic cord as far proximally as the internal ring (21, 22). Jarow et al., 1992, examined the spermatic cords using loupe magnification for 12 men who underwent inguinal varicocelectomy and reported 1–3 (mean of 2) testicular arteries within the inguinal spermatic cord (23). Hopps et al., 2003; identified two arteries in 42% of all dissections and three arteries in 33% in the spermatic cords during microsurgical varicocelectomy at the sub-inguinal level (24). Several surgical techniques have been described, including the Palomo operation (ligation of internal spermatic veins) in the retroperitoneal space (25), modified Palomo operation (ligation of the vascular pedicle above the vas deferens) by opening the external oblique aponeurosis (26), and the sub-inguinal approach of Ivanissevich (ligation of the vascular pedicle at the superficial inguinal ring) without opening the external oblique aponeurosis (27). Loupes or an operating microscope is used for optical magnification by most experts who perform inguinal or sub-inguinal surgical repair, and this technique maximizes preservation of arterial and lymphatic vessels while reducing the risk of persistence or recurrence of varicocele (28).

## 2. Objectives

The aim of this study was to evaluate the outcome of varicocelectomy using a modified microsurgical method, specifically a loupe-assisted method, and its effects on sperm parameters in infertile men.

## 3. Patients and Methods

### 3.1. Protocol

During a 3-year period, 40 patients with varicocele and primary infertility were included in this prospective study. Half of the patients (20 patients) were treated by a sub-inguinal approach assisted with loupe magnification (Group A) and the other half (20 patients) by the same approach but without magnification (Group B). Written consent was obtained from all patients after an explanation was provided regarding the nature of operation. Routine preoperative investigations were performed and included the complete blood count (CBC), blood glucose, liver function test (LFT), renal function test (RF), coagulation profile, blood grouping and hepatitis B and C antibodies. Exclusion criteria included previous inguinal or scrotal surgery (varicocelectomy, cryptorchidism or hernia repair), secondary infertility, azoospermia and female factors or any finding contraindicated for surgery. All of the patients presented with infertility for least 1 year without history of any medical treatment that can affect sperm parameters for at least 3 months prior to this study. Varicocele was diagnosed clinically and further confirmed using color Doppler ultrasound.

Physical examination, semen analysis and hormonal evaluation including FSH, LH, total testosterone and prolactin hormones were performed for each patient. The physical examination (left or right, unilateral or bilateral varicocele) and the grade (Grade I to III) of varicocele were determined by inspection and palpation with the patient in an upright position. The grades of varicocele were classified by using various methods including physical examination and confirmed by scrotal ultrasound and Doppler examination. The criteria were: Grade I (small), detected by palpation with difficulty but increased by Valsalva’s maneuver; Grade II (moderate), detected easily by palpation without Valsalva’s maneuver; Grade III (large), detected visually at a distance.

At least three semen samples were collected by masturbation after 3 days of abstinence and used for preoperative semen analysis according to World Health Organization guideline 1999 for each patient. All patients were examined at 3 and 6 months postoperatively, which included semen analyses for the assessment of sperm concentration, motility and abnormal morphology of spermatozoa, hormonal profile, scrotal ultrasound, and color Doppler. The recurrence of varicocele, hydrocele and or any other complications after surgical correction of varicocele was regularly assessed.

### 3.2. Surgical Technique

In group A, the patient was placed in the supine position under spinal anesthesia. The incision was made transversely with a length of approximately 2 to 2.5 cm at the level of external inguinal ring, just outside the pubic tubercle. The external ring was not opened; therefore, the inguinal canal was kept intact. By retracting the edges of the wound, the spermatic cord could be identified by the appearance of the blue color of the spermatic veins. After loosening of the spermatic cord by moving it medially and laterally, the cord could be looped and then easily externalized on a vascular tape without tension. The tissues external to the spermatic cord were examined first for any engorged veins; if present, they were ligated accordingly. The external and intermediate spermatic fascia were opened to expose the internal spermatic veins and fat. After the internal spermatic fascia of the spermatic cord was opened, the dissection was continued with the aid of a×3.0 loupe. Lymphatics were characterized by their crystal clear intravascular contents. The arteries were identified by their clearly visible pulsations. The engorged internal spermatic veins were identified and dissected carefully with mosquito clamps. Manipulating the mosquito clamps under the target vessel by a gentle up-and-down movement helped to differentiate a vein from an artery or a lymphatic vessel. While the vessel was isolated, a loop of 3-0 Vicryl was passed beneath, and then the loop end was divided to make double ties. The vessel was ligated at both ends and severed with sharp scissors. The compartment of the vas deferens was protected and left untouched except when abnormally engorged veins were evident. After the procedures performed inside the spermatic cord were completed, the wound was closed subcutically with 4-0 Vicryl sutures.

The technique mentioned above was also performed for the Group B patients but without the aid of the magnifying loupe. In general, the testicle was not delivered from the wound; therefore, the gubernacular veins were not touched. The patients were discharged in the next morning. The semen parameter data are presented as the mean standard deviation (SD). P < 0.05 was considered significant.

## 4. Results

In total, 40 patients were included in this study. The age of the patients varied from 25 to 38 years (mean 32.5). Regarding the varicocele grading, 20 patients (50%) were grade III, 12 patients (30%) were grade II and 8 patients (20%) were grade I. In 38 patients (95%), the varicocele was on the left side, whereas it was on the right side in two patients (5%). All patients presented with subfertility, but two patients (5%) also complained of intolerable pain, and five patients (12.5%) with visible deformity in addition to infertility (Table 1).

**Table 1:** Presenting Symptom

**Symptoms**	**Number**	**%**
Subfertility	40	100
Pain	2	5

When comparing pre-operative and post-operative semen parameters in Group A, there was a significant increase in the sperm concentration and in the percentage of motile spermatozoa, as well as significant reduction in spermatozoa with abnormal morphology, as early as the third month after varicocelectomy Table 2.The three parameters became normal during the following three months. In Group B, there was an increase in the sperm concentration among the motile spermatozoa as well as a reduction in the spermatozoa with abnormal morphology, but without significant differences. There was a significant postoperative increase in the level of testosterone in both groups, but the other hormones (FSH, LH and practin) remained unchanged, as shown in Table 3.

**Table 2:** Pre and Post-Operative Semen Analysis in Both Groups

**Operation**	**Sperm Parameters, Mean ± SD**	**Before Treatment, Mean ± SD**	**3 Months After Treatment, Mean ± SD**	***P***	**Sig.**	**6 Months After Treatment, Mean ± SD**	***P***
Group (A) Loupe-assisted varicocelectomy							
	Sperm count, × 10^6^/mL	15 ± 5	35 ± 10	< 0.0001	S a	37 ± 11	< 0.0001
	Sperm motility %	24 ± 8	45 ± 14	< 0.0001	S	48 ± 15	< 0.0001
	Abnormal sperm morphology %	54 ± 17	30 ± 9	< 0.0001	S	29±8	< 0.0001
Group (B) Varicocelectomy without loupe assistance							
	Sperm count, × 10^6^/mL	17 ± 6	20 ± 6	0.12	NS a	21 ± 9	0.11
	Sperm motility %	25 ± 8	30 ± 10	0.09	NS	32± 15	0.07
	Abnormal sperm morphology %	51 ± 15	45 ± 14	0.20	NS	43 ± 13	0.08

^a^Abbreviations: NS, not significant; S, significant

**Table 3:** Pre and Postoperative Hormonal Levels in Both Groups

**Hormones**	**Preoperative, Mean ± SD**	**3 Months Postoperative, Mean ± SD**	***P***	**Sig,**	**6 Months Postoperative, Mean ± SD**	***P***
Testosterone, mmol/L	10.2 ± 3	18.9 ± 7	< 0.0001	S a	19.4 ± 8	< 0.0001
FSH, IU/L	12 ± 4	11 ± 3.5	0.24	NS a	12 ± 3	1
LH, IU/L	3.1 ± 1	3.5 ± 1.3	0.13	NS	3.2 ± 1.5	0.08
Prolactin, mlU/L	60 ± 16	61 ± 13	0.76	NS	61 ± 14	0.77

^a^Abbreviations: NS, non significant; S, significant

Regarding post-operative complications, in Group A only one patient (5%) developed scrotal hematoma and two (10%) developed wound infection, whereas in Group (B) the complication rate was higher, with two patients (10%) developing scrotal hematoma, two (10%) developing wound infection, three (15%) developing hydrocele, two (10%) developing recurrence, and two (10%) developing scrotal edema as shown in Table 4.

**Table 4:** Post-Operative Complications

**Complication**	**Group A, No.(%) (n = 20)**	**Group B, No.(%) (n = 20)**
Scrotal hematoma	1 (5)	2 (10)
Wound infection	2 (10)	2 (10)
Hydrocele	-	3 (15)
Recurrence	-	2 (10)
Scrotal edema	-	2 (10)

## 5. Discussion

The association between clinical varicocele and impaired spermatogenesis is well described (29). Varicocele is the most frequently observed surgically correctable cause of male infertility (30). The exact pathophysiology of varicocele remains unknown, but it has been previously reported that the reflux of renal prostaglandins may underlie the testicular injury (31). Recent studies on the mechanism of varicocele-induced infertility note an increase in testicular temperature caused by the impairment of the countercurrent heat exchange mechanism (32). Subfertile men with varicoceles usually present with asthenospermia, teratospermia, oligospermia, or combinations of these features, and varicocelectomy is usually indicated, but it is not possible to predict who will ultimately benefit. Improvement in the quality of semen occurred in 51–74% of the patients and the pregnancy rate increased to 24–71% after varicocelectomy, whereas others have found no beneficial effect of varicocelectomy on pregnancy rates or semen quality (33).

Varicocele can be treated by a routine surgical intervention (varicocelectomy), microsurgery varicocelectomy, which is considered as the gold standard approach to varicocele repair (19), or by radiological embolization (34). Routine varicocelectomy is still the most popular treatment, even in the era of assisted reproductive techniques when treatment at the gamete level is feasible (35). Different outcomes, including increased pregnancy rate or improvement in one, two, or all the three seminal parameters have been used to evaluate the success rate of varicocelectomy (36). In a review of varicocele repair, Ficarra et al. (2006) they found a significant increase in the pregnancy rate of patients who underwent varicocele treatment (36.4%) compared with patients who received no treatment (20%) (15). In another study by Marmar et al. (2007), the pregnancy rate in patients who underwent surgical varicocelectomy was 33% as compared to 15.5% in the controlled patients who received no varicocelectomy (16).

Watanabe et al. (2005) stated that the sub-inguinal microscopic procedure is a minimally invasive varicocelectomy technique because of its postoperative mobility and is an effective treatment for infertile men with left clinical varicocele (37). Several studies indicate that larger varicoceles are associated with greater impairment of spermatogenesis (38), whereas others suggest that varicocele size does not correlate with the response to surgery (39). It is proposed that the varicocele must be treated when all of the following conditions are present: the couple’s infertility is documented, the varicocele is palpable, there is no incurable infertility problem in the female, and at least one abnormality is present in the semen analysis (40).

During the past several decades, many different approaches or tools have been used for the treatment of varicocele with varying rates of success and complications. The best treatment modality for varicocele can be selected only after comparing the recurrence rate, improvement in semen parameters, and complication rates of these approaches (41). Recently, the sub-inguinal varicocelectomy, which was first described by Marmar (16), has become more popular because sub-inguinal varicocelectomy has a lower incidence of morbidity, complications, and residual lesions. However, this procedure reveals many more tedious small veins. Therefore, the need for more sophisticated microsurgical techniques steepens the learning curve (42). The high success rate of sperm recovery may be attributed to the preservation of the testicular artery and lymphatics (28). Although the necessity of preserving the testicular artery remains controversial, there are many reports of testicular atrophy following non-microsurgical conventional varicocelectomy or blind cord block only (43, 44). Possible adverse effects of hydrocele were reported by Szabo and Kessler (45). Postoperative hydrocele is highly correlated with varicocelectomy. In fact, the testicular artery and lymphatics can be accurately preserved by microsurgical varicocelectomy (46).

In our work, the complication rate of postoperative hydrocele was 0%, which is superior to that of the conventional procedure. Reported incidences of postoperative hydrocele are between 7% and 30% (47, 48). Abdel-Magidand Othman (2010) reported a postoperative hydrocele complication rate of 1.2% in the microsurgical subinguinal varicocelectomy group and 33.8% in the non-magnified subinguinal varicocelectomy group (49). Another factor influencing the empirical outcome is the recurrence of postoperative varicocele. The usual general recurrence rate for varicocelectomy ranges from 15% to 25% (45, 48). However, we had no recurrence in our study. The effect of varicoceles on sperm production alters spermatogenesis and often can result in the generalized impairment of sperm production, which is characterized by decreased sperm density and motility and an increase in immature spermatozoa ranging from oligozoospermia to complete azoospermia (50).

In the present study, there was a statistically significant increase in the sperm concentration and in the percentage of motile spermatozoa, as well as a significant reduction in the spermatozoa with abnormal morphology, as early as the third month after varicocelectomy. The three parameters became normal during the following 3 months, and this result is in agreement with the studies of Masanobu et al. (1996) (51), Cozzolino et al. (2001) (33), and Shamsa et al. (2010) (52). There was a significant increase in the postoperative level of testosterone but the other hormones (FSH, LH, and practin) showed no effect. This result is in agreement with studies by Cayan et al. (1999) (53); Podesta et al. (1994) (54) and Onozawa et al. (2002) (5). Varicocelectomy probably has positive effects on Leydig cell function. Defective testosterone synthesis has been reported to be associated with varicocele (55), probably through intratesticular hyperthermia, which inhibits 17a-hydroxyprogesterone aldolase, an enzyme responsible for the conversion of 17a-hydroxyprogesterone to testosterone. Thus, Leydig cell function and serum free testosterone levels should be improved on removing the inhibition of 17a-hydroxyprogesterone aldolase by relieving intratesticular hyperthermia through varicocelectomy (56).

Varicocele is a common cause of infertility and is a curable disease in patients. Loupe-assisted sub-inguinal varicocelectomy provides a significant improvement in sperm parameters and is a safe, simple, and effective method for the treatment of sub-fertile men, especially in medical facilities without microscopic equipment.
